# ParallABEL: an R library for generalized parallelization of genome-wide association studies

**DOI:** 10.1186/1471-2105-11-217

**Published:** 2010-04-29

**Authors:** Unitsa Sangket, Surakameth Mahasirimongkol, Wasun Chantratita, Pichaya Tandayya, Yurii S Aulchenko

**Affiliations:** 1 Center for Genomics and Bioinformatics Research, Faculty of Science, Prince of Songkla University, Songkhla, 90112, Thailand; 2 Medical Genetic Section, National Institute of Health, Department of Medical Sciences, Ministry of Public Health, Nonthaburi, 11000, Thailand; 3 Department of Pathology, Faculty of Medicine, Ramathibodhi Hospital, Mahidol University, Bangkok, 10400, Thailand; 4 Department of Computer Engineering, Faculty of Engineering, Prince of Songkla University, Songkhla, 90112, Thailand; 5 Department of Epidemiology, Erasmus MC Rotterdam, Postbus 2040, 3000 CA Rotterdam, the Netherlands; 6 Quantitative Integrative Genomics Group, Institute of Cytology & Genetics SD RAS, Novosibirsk 630090, Russia

## Abstract

**Background:**

Genome-Wide Association (GWA) analysis is a powerful method for identifying loci associated with complex traits and drug response. Parts of GWA analyses, especially those involving thousands of individuals and consuming hours to months, will benefit from parallel computation. It is arduous acquiring the necessary programming skills to correctly partition and distribute data, control and monitor tasks on clustered computers, and merge output files.

**Results:**

Most components of GWA analysis can be divided into four groups based on the types of input data and statistical outputs. The first group contains statistics computed for a particular Single Nucleotide Polymorphism (SNP), or trait, such as SNP characterization statistics or association test statistics. The input data of this group includes the SNPs/traits. The second group concerns statistics characterizing an individual in a study, for example, the summary statistics of genotype quality for each sample. The input data of this group includes individuals. The third group consists of pair-wise statistics derived from analyses between each pair of individuals in the study, for example genome-wide identity-by-state or genomic kinship analyses. The input data of this group includes pairs of SNPs/traits. The final group concerns pair-wise statistics derived for pairs of SNPs, such as the linkage disequilibrium characterisation. The input data of this group includes pairs of individuals. We developed the ParallABEL library, which utilizes the Rmpi library, to parallelize these four types of computations. ParallABEL library is not only aimed at GenABEL, but may also be employed to parallelize various GWA packages in R. The data set from the North American Rheumatoid Arthritis Consortium (NARAC) includes 2,062 individuals with 545,080, SNPs' genotyping, was used to measure ParallABEL performance. Almost perfect speed-up was achieved for many types of analyses. For example, the computing time for the identity-by-state matrix was linearly reduced from approximately eight hours to one hour when ParallABEL employed eight processors.

**Conclusions:**

Executing genome-wide association analysis using the ParallABEL library on a computer cluster is an effective way to boost performance, and simplify the parallelization of GWA studies. ParallABEL is a user-friendly parallelization of GenABEL.

## Background

GWA analysis [1] is a well established and powerful method for identifying loci associated with variations of complex genetic traits such as common diseases. Hundreds of new genes have been implicated in human health and disease during the last few years in various GWA studies[2]. In a typical study, hundreds of thousands, or millions, of single-nucleotide polymorphisms (SNPs) are typed in thousands of individuals in order to detect genetic risk factors.

GenABEL is a specialized library package for GWA analysis [3] implemented in R, an open source statistics programming language and environment [4,5]. GenABEL enables GWA analysis to be done using a regular desktop computer due to its efficient data storage and memory management. Nevertheless, analysis of very large data sets are computationally challenging and may take hours or weeks to complete. Examples include the utilization of sophisticated adjustments for population stratification and relationship structures, the estimation of linkage disequilibriums and the calculation of genome-wide identity-by-state, haplotypic tests, and permutation analyses.

To increase the computational throughput, a user can partition their data into sets, and perform the analysis of the sets across a network of computers; a concept known as parallel and/or distributed computing. However, performing such analysis requires high levels of computer expertise. The user needs sufficient programming skills to partition and distribute data, control and monitor tasks across the computers, and merge output files. Occasionally, a data set may fail to be processed, e.g. if the user did not partition the data into small enough subsets to be processed on a particular machine. Also, the outputs from the computers may be scattered and their order hard to follow.

**Figure 1:**
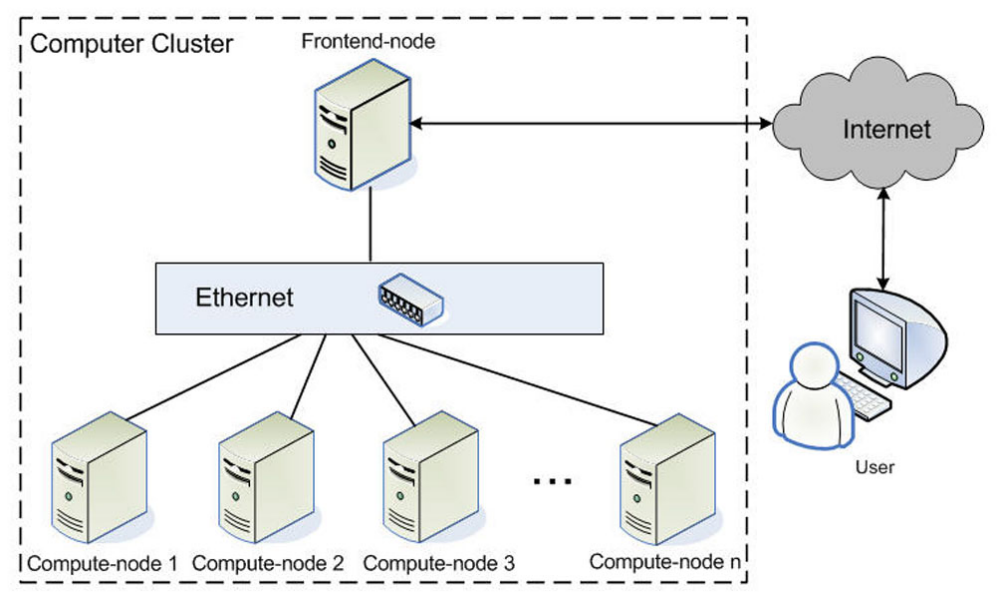
**Computer Cluster Architecture**. The user can submit tasks to the cluster of computers via the Internet. Once the user submits a job to the computer cluster, the frontend-node schedules and distributes the smaller partitioned tasks to the compute-nodes. The output from each compute-node is merged by the frontend-node.

Parallel computing is an intuitive, and powerful, method for increasing computational throughput. A task is separated into smaller tasks, and each is processed independently, in parallel, using multiple Central Processing Units (CPUs) or a cluster of computers. The outputs from each task must later be merged [6]. A general architecture for parallel computing is shown at Figure 1. Most tasks solved in GWA analysis are suitable for parallelization, due to their computational independency, with parallelization achieved at the data level. For example, association tests can usually be done separately for each SNP and/or a small group of SNPs. Consequently, parallelization is a beneficial way to reduce the computing time, with few overheads incurved in large-scale GWA analyses.

Several attempts had been made to parallelize genetic association analyses. Grid Engine, a cutting-edge parallel tool, can schedule parallel tasks involving genetic association analysis programs [7] such as FBAT [8] and UNPHASED [9]. The approach, first proposed by Mishima et al., is based on non-parallel code combined through process-based parallelization. The downside is that the user still needs to monitor when each task is finished, and when the outputs from all the tasks can be merged. Moreover, each process may take a very long time to finish, and load balance can be problematic. A granularity problem (a high computation to communication ratio) may occur, but higher power compute-nodes or code parallelization are possible solutions. The R/parallel package has been used to automate loop parallel execution, but the application must run on a single computer with multi-core processors, and does not currently support cluster computing [10]. Its inclusion would allow the computing time limit of the package to be eliminated. Misawa and Kamatani [11] developed the ParaHaplo package for haplotype-based whole-genome association studies using parallel computing. It is aimed at correcting multiple comparisons in multiple SNP loci in linkage disequilibrium. There are other statistical analyses requirements in GWA studies, such as obtaining statistics for a particular SNP or a trait, association test, characterizing an individual in the study, and pair-wise statistics between individuals. Furthermore, Ma et al. [12] developed EPISNPmpi, a parallel system for epistasis testing in large scale GWA analysis.

Rmpi [13] is an R library which provides various functions to parallelize tasks on R using the MPI (Message-Passing Interface) [14]. Rmpi employs various functions to manage flow analysis in parallel environment, and is applicable for employing multi-core CPUs distributed across many computers, not only multi-core CPUs on a single computer. However, it is difficult, if not impossible, for a non-programmer to write a parallel Rmpi program. Therefore, SPRINT [15] was developed to implement parallel R functions. Although users can use SPRINT easily, it does not specifically support GWA studies.

In this article, we present the development of our ParallABEL library, a new R library for parallelization of GWA studies based on Rmpi. ParallABEL aims to speed up the computation of GWA studies for various statistical analysis requirements and also simplify analysis parallelization. With ParallABEL, the users do not need to be experts programming on partitioning and distributing data, controling and monitoring tasks, and merging output files.

## Implementation

### GWA Function Grouping

Statistical analyses in GWA studies can be categorized into four groups based on the nature of the statistics computed and type of data used. These four groups can be parallelized in distinct ways. Table 1 shows the name and description of the GenABEL function in each group. The first group contains statistics computed for a particular SNP, or a trait, such as the SNP characterization statistics (e.g. call rates, HWE testing), produced by GenABEL's *summary.snp.data *or association test statistics (the *qtscore, mlreg *and *mmscore *GenABEL functions). The second group holds statistics characterizing an individual in the study, such as, summary statistics of genotype quality for each sample (obtained with the GenABEL *perid.summary *and *hom *GenABEL functions). The third group consists of pair-wise statistics derived from analyses between each pair of individuals in the study, including genome-wide identity-by-state and genomic kinship analyses. This is one of the most computationally intensive analyses, obtained through GenABEL's *ibs *function. The final group concerns pair-wise statistics derived for pairs of SNPs, such as linkage disequilibrium characterisation (the *dprfast, rhofast *and *r2fast *functions). While the number of SNP pairs is generally very large, analyses are usually limited by their pair-wise physical distance, making them less demanding than pair-wise individual analyses, such as IBS computations.

**Table 1:** GWA analyses grouping

function name of GenABEL	Description	group
summary.snp.data	Provides summary of observed genotypes, allelic frequency, genotypic distribution, P-value of the exact test for HWE and chromosome	1
qtscore	Fast score test for association between a trait and genetic polymorphism	1
mlreg	Linear and logistic regression and Cox models for genome-wide SNP data	1
mmscore	Score test for association between a trait and genetic polymorphism, in samples of related individuals	1
perid.summary	Produces call rate and heterozygosity per person	2
hom	Computes average homozygosity (inbreeding) for a set of people, across multiple markers. Can be used for Quality Control (e.g. contamination checks)	2
ibs	Given a set of SNPs, computes a matrix of average IBS for a group of people	3
dprfast	Given a set of SNPs, computes a matrix of D'	4
rhofast	Given a set of SNPs, computes a matrix of rho	4
r2fast	Given a set of SNPs, computes a matrix of r2	4

The name and description of function of GenABEL in each group

We have developed the ParallABEL library to parallelize the serial functions of these groups using Rmpi library. The four implementation groups are named Type1_parall_by_SNPs for the first group, Type2_parall_by_individuals for the second group, Type3_parall_by_pairs_of_individuals for the third group and Type4_parall_by_pairs_of_SNPs for the fourth group.

### Data Partitioning

An advantage of ParallABEL is usage simplicity, hiding otherwise tedious scripts for file management monitoring tools. These functions not only partition input data with automatic load balancing, but also gather output from each processor automatically. Load balancing is critical because an unbalanced work load will result in higher loads for particular processors, which eventually undermines the overall performance.

**Figure 2:**
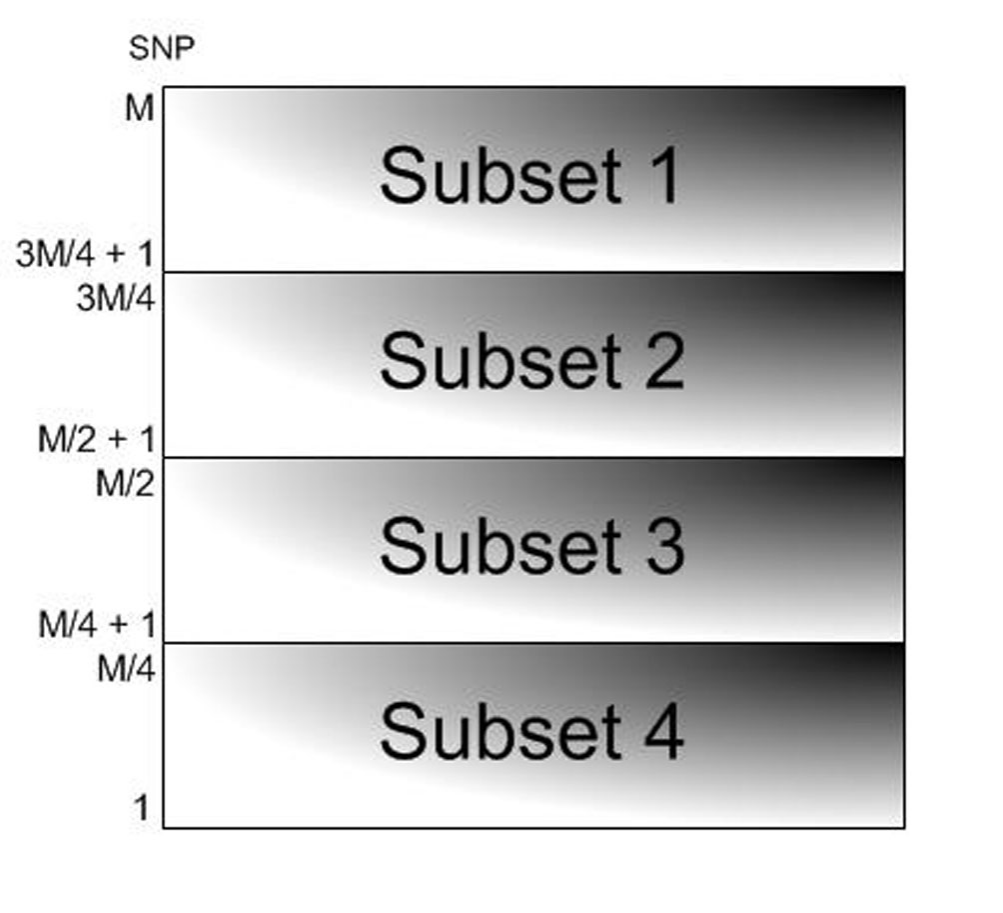
**Data partitioning for Type1_parall_by_SNPs**. Data partitioning for Type1_parall_by_SNPs Type2_parall_by_individuals when *M *= 800 and *P *= 4.

The input data of Type1_parall_by_SNPs contains SNPs equally partitioned into *P *subsets (where *P *is the number of available processors). If the number of SNPs is *M*, the number of SNPs in a subset is:

If there are *M *SNPs and 4 processors, the SNPs will be partitioned into 4 smaller subsets. Each containing *M*/4 SNPs as shown in Figure 2. However, the last subset to be generated may contain more SNPs than others, caused by integer division. For example, if there are 801 SNPs and 4 processors, Subset 1 to Subset 3 will contain 200 SNPs, but Subset 4 will have 201. The SNPs in each subset will execute on separate processors.

The input data for Type2_parall_by_individuals consists of individuals, partitioned like Type1_parall_by_SNPs

**Figure 3:**
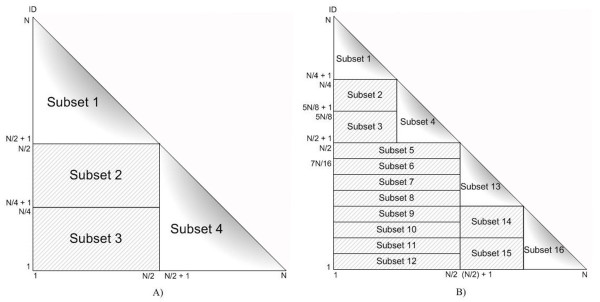
**Data partitioning for Type3_parall_by_pairs_of_individuals**. A) The first data partitioning for Type3_parall_by_pairs_of_individuals when the number of individuals = *N*. There are 4 equal subsets. B) The second data partitioning for Type3_parall_by_pairs_of_individuals when the number of individuals = *N*. There are 16 equal subsets.

The input data for Type3_parall_by_pairs_of_individuals is a pair of individuals, and performs a more complicated partitioning than Type1_parall_by_SNPs and Type2_parall_by_individuals. The data is divided until the number of processors is equal to, or less than, the number of subsets for load balancing on each processor. If the number of processors is equal to the number of subsets, then each processor executes an individual pair of each subset. If the number of processors is less than the number of subsets, then each processor executes an equal number of individual pairs (where possible). Figure 3 shows Type3_parall_by_pairs_of_individuals with *N *individuals. The statistics is calculated from the cross operation of an individual in a row with an individual in a column. The input data is partitioned into 4 subsets using the data partitioning shown in Figure 3A. However, if the number of processors is more than 4, the subsets are partitioned again. Subset 1 and Subset 4 are split into 8 subsets during the first stage of the data partitioning, while Subset 2 and Subset 3 are divided into 8 subsets by row, as shown in Figure 3B. There are 16 subsets altogether in the second stage of the data partitioning.

The SNPs input of Type4_parall_by_pairs_of_SNPs will be partitioned in a similar way to Type3_parall_by_pairs_of_individuals.

### Implementation

**Figure 4:**
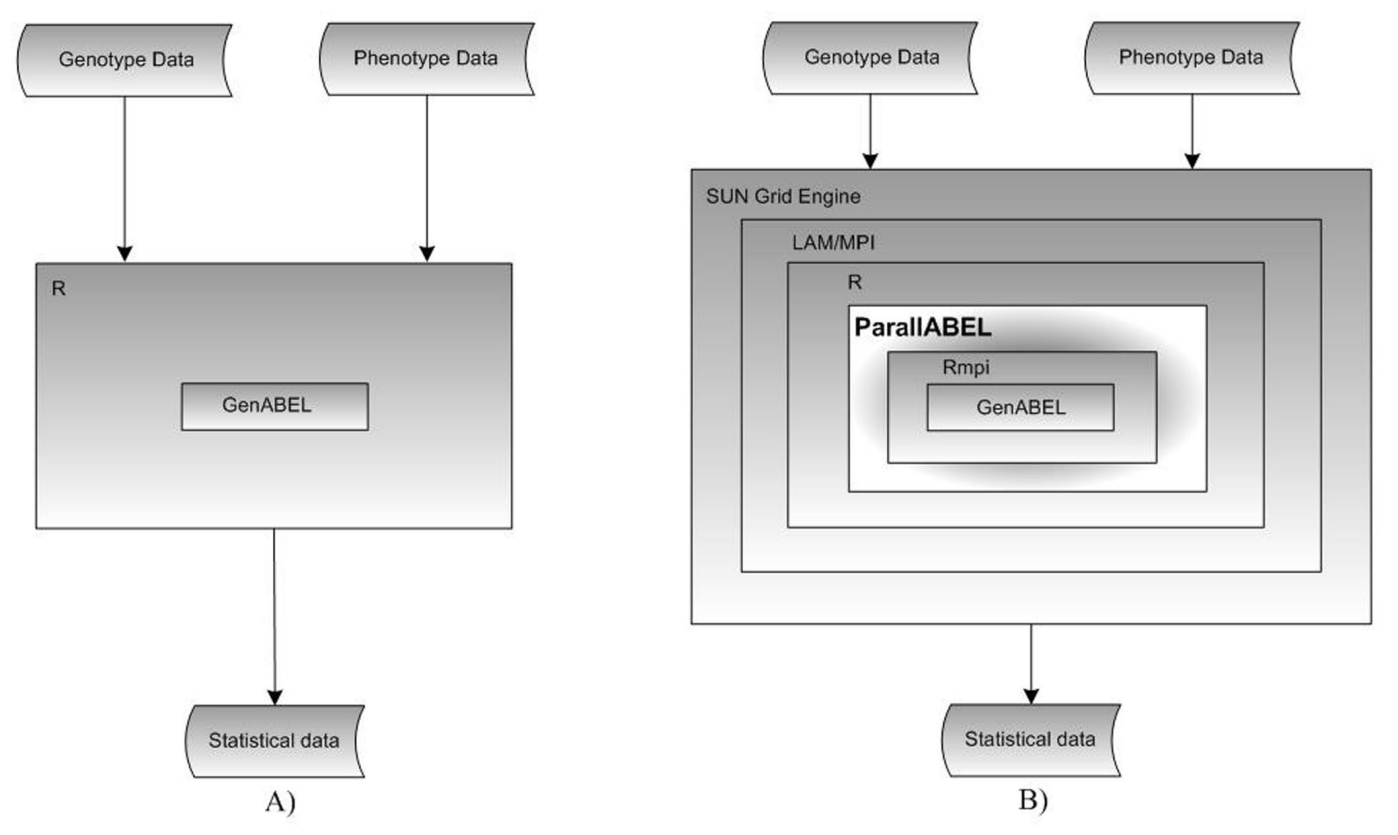
**GWA Computing Workflow**. A) Sequential GWA Computing Workflow, which runs on a single processor or computer. B) Parallel GWA Computing Workflow which runs on a multiprocessor or a set of computers.

The workflow for GWA analysis on a single processor or computer is presented in Figure 4A. This workflow runs. The genotype and phenotype data is processed by the GenABEL library, which works under the R program. GenABEL sequentially processes the raw data, producing statistical data as its outputs.

This sequential workflow may take a very long time to produce some demanding statistical analyses. Our novel parallel workflow for producing statistical data in GWA studies is shown in Figure 4B, and can save computing time. The genotype and phenotype data is passed for distribution to the SUN Grid Engine, a job scheduler. It queues jobs and assigns them to processors in a cluster. LAM/MPI (Local Area Multicomputer/Message Passing Interface) [16] has various functions which can be called by Rmpi to parallelize R. ParallABEL parallelizes GenABEL using this Rmpi library. The statistical data from this workflow has been validated by comparing it with the outputs from the non-parallel approach. ParallABEL runs not only on Linux cluster, such as the Rocks Cluster Distribution, but also on any Operating System that supports R and LAM/MPI or Open MPI, such as the Unix and Solaris Operating Systems. It can also run on computer clusters lacking the Sun Grid Engine by executing immediately. However, the administrator will normally not allow a user to run a parallel program without utilizing a queuing process from the Sun Grid Engine.

To parallelize GWA studies, ParallABEL running on the frontend-node partitions input data into smaller subsets so that tasks can be fairly distributed among the processors. It sends tasks to idle processors on compute-nodes. When the computation on a compute-node has finished, the frontend-node will send another task to the idle processor - a cycle that continues until all the tasks are completed, which is known as the 'task pull' method [17]. When all the tasks are finished, the frontend-node automatically merges all the outputs.

**Figure 5:**
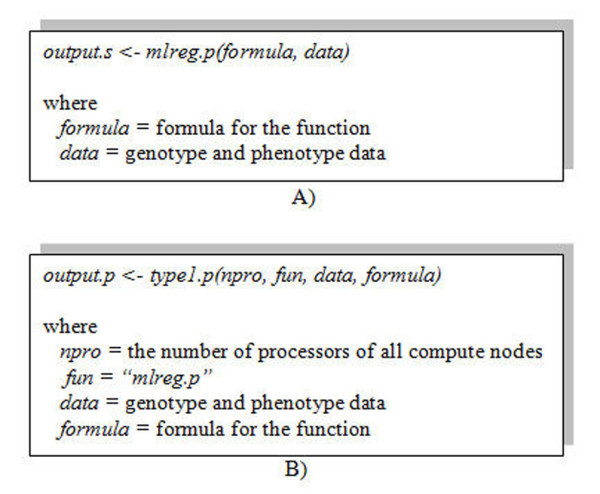
**A comparison of using a sequential and parallel function**. A) Executing the *mlreg.p *function sequentially on a processor B) Parallelizing the *mlreg.p *function on more than one processor. The user supplies the function name and number of processors to the parallel function.

**Figure 6:**
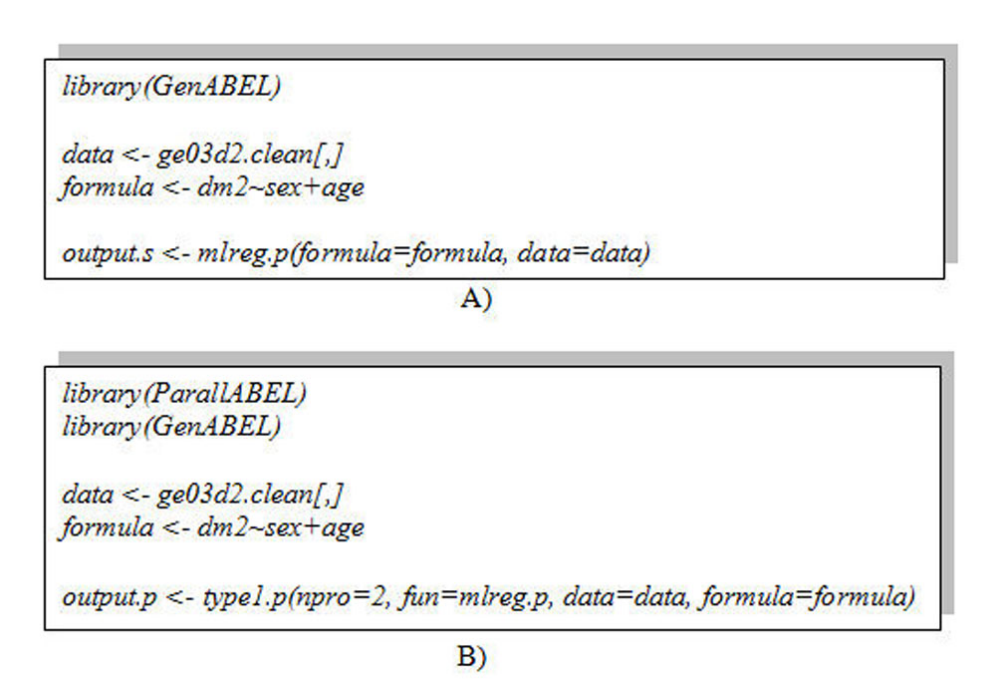
**A comparison execute sequential and parallel function**. A) Execute the *mlreg.p *function sequentially on a processor B) Parallelize the *mlreg.p *function on more than one processor.

Users can use ParallABEL to parallelize GenABEL GWA functions as easily as using GenABEL for sequential analyses. An example of the *mlreg.p *command sequentially on a processor is shown in Figures 5A and 6A. The executing command that parallelizes *mlreg.p *to run on multiple processors using Type1_parall_by_SNPs is shown in Figures 5B and 6B.

## Results

Our computer cluster, Hanuman, runs Rocks Cluster Distribution version 4.3, which includes the SUN Grid Engine version 4.3 [18]. The cluster consists of 5 IBM servers xSeries 336s, comprising of a frontend-node and four compute-nodes. All servers have 2 SINGLE-CORE Intel Xeon (2.8 GHz) processors and 4 GB RAM. The frontend-node and all the compute-nodes are connected through an Ethernet switch, and the user can connect via the Internet. The cluster provides LAM/MPI version 7.1.2, R program version 2.8.1, Rmpi library version 0.5-6, and GenABEL version 1.4-2, which are utilized as components by our ParallABEL library.

The North American Rheumatoid Arthritis Consortium (NARAC) data is part of a dataset employed to observe associations between disease and variants in the major-histocompatibility-complex locus [19]. The NARAC genotype data contains 545,080 SNPs from 2,062 individuals. The data was used to measure the performance of ParallABEL by employing 868 individuals for cases, and 1,194 individuals as controls.

**Figure 7:**
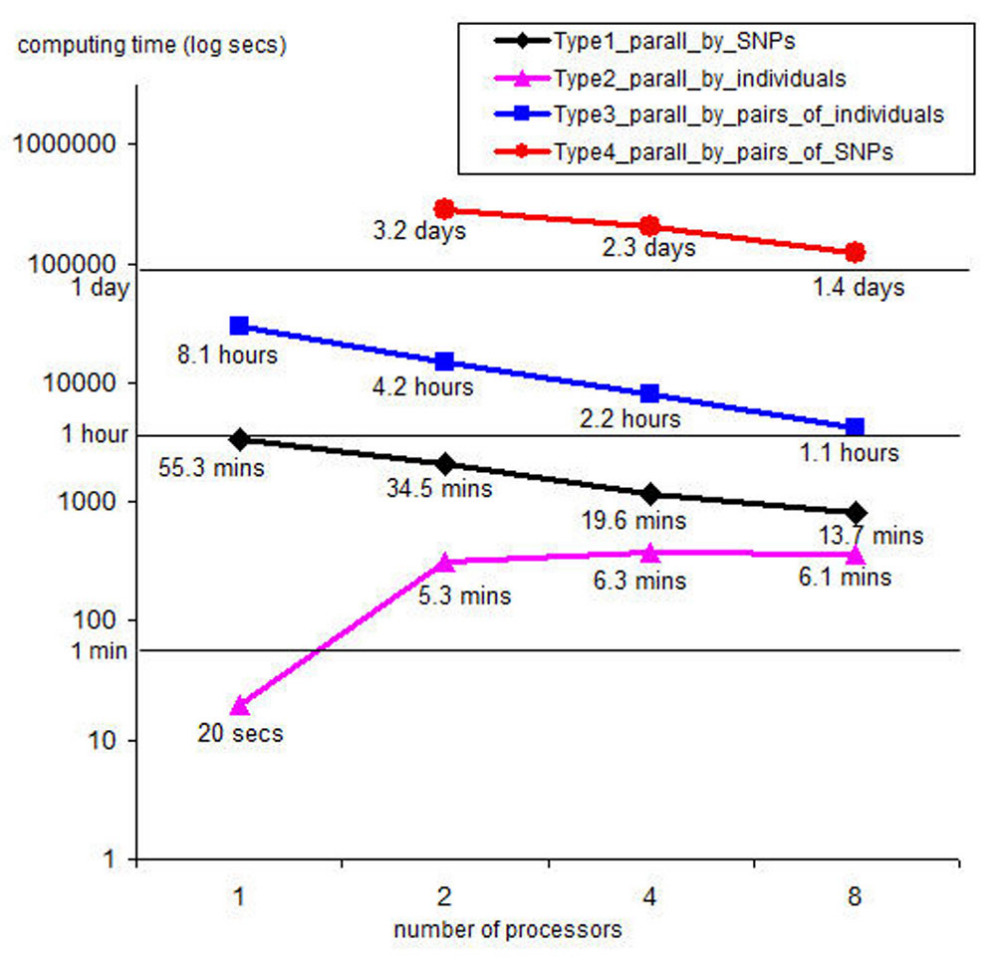
**Trace results from ParallABEL for NARAC data**. Trace results from Type1_parall_by_SNPs, Type2_parall_by_individuals, Type3_parall_by_pairs_of_individuals, and Type4_parall_by_pairs_of_SNPs for NARAC data. When Type1_parall_by_SNPs is executed by the GenABEL *mlreg *function, Type2_parall_by_individuals is executed by the GenABEL *hom *function, Type3_parall_by_pairs_of_individuals is executed by the GenABEL *ibs *function, and Type4_parall_by_pairs_of_SNPs is executed by the GenABEL *r2fast *function. If there is only one processor, then the data is analysed using GenABEL. If there is more than one processor, the data is analysed using ParallABEL package.

Trace results from Type1_parall_by_SNPs, Type2_parall_by_individuals, Type3_parall_by_pairs_of_individuals, and Type4_parall_by_pairs_of_SNPs for the NARAC data are shown in Figure 7. Type1_parall_by_SNPs was executed by the GenABEL *mlreg *function, Type2_parall_by_individuals was executed by the GenABEL *hom *function, Type3_parall_by_pairs_of_individuals was executed by the GenABEL *ibs *function, and Type4_parall_by_pairs_of_SNPs was executed by the GenABEL *r2fast *function.

ParallABEL reduced the computing time for Type3_parall_by_pairs_of_individuals, especially with 8 processors. The Type3_parall_by_pairs_of_individuals executing speed on eight processors was approximately seven times faster than on one processor. On a single processor, the complete analysis took 8.1 hours, but only 1.1 hours with 8 processors. The computing time for Type1_parall_by_SNPs also tends to be like that for Type3_parall_by_pairs_of_individuals.

The computing time for the sequential version of Type2_parall_by_individuals can be very short (e.g. 20 seconds). While the parallel version took longer (5.3 minutes for 2 processors), due to the overhead of data partitioning, data distribution, and data merging. Data distribution can be time consuming because the data must be saved on the frontend-node before the compute-nodes can load it, and the frontend-node must also speed time communicating with the compute-nodes. In addition, GenABEL is tailored to quickly retrieve subset of SNPs, as this is a typical GWA scan procedure, but is much less efficient in retrieving subsets of individuals, which is less typical. Thus the overhead of data partitioning in subsets of individuals prevailed over the gain achieved by parallel processing. These results highlighted a place where GenABEL data storage and processing is ineffective, and we are currently working on better algorithms to do by-individual analyses.

Type4_parall_by_pairs_of_SNPs was executed by the GenABEL *r2fast *function. A single processor can not pass all the SNPs in the NARAC data to *r2fast *due to CPU memory limitations so, the analysis was done separately for each chromosome. Even then, a single processor can not call *r2fast *with a chromosome with more than 10,000 SNPs, which affects 20 chromosomes in the data. However, ParallABEL can run *r2fast *with a chromosome with more than 10,000 SNPs by employing a set of processors. The chromosome data is automatically partitioned based on the number of SNPs, as shown in Table 2. If the number of SNPs for a chromosome is between 11 and 14,000, then the data is partitioned into at least 4 balanced subsets. If the number of the SNPs is between 14,001 and 28,000, then the data is divided into at least 16 balanced subsets. If the number of SNPs is between 28,001 and 65,000, then the data is split into at least 64 balanced subsets. The data is automatically partitioned until the number of processors is equal to, or less than, the number of subsets for load balancing on each processor. The trace example results for Type4_parall_by_pairs_of_SNPs of NARAC data are shown in Figure 7.

**Table 2:** Data partitioning for each chromosome

Chromosome name	Number of SNPs	Number of subsets
19,20,21,22, X, Y	11-14,000	4
9,11,12,13,14,15,16,17,18	14,001-28,000	16
1,2,3,4,5,6,7,8,10	28,001-56,000	64

The least number of subsets of each chromosome partitioned by the number of SNPs

Type4_parall_by_pairs_of_SNPs took only 1.4 days to execute on eight processors, indicating that time-saving with ParallABEL is linearly correlated to the number of nodes. This suggests that with more SNPs, more computing time will be saved by ParallABEL.

**Figure 8:**
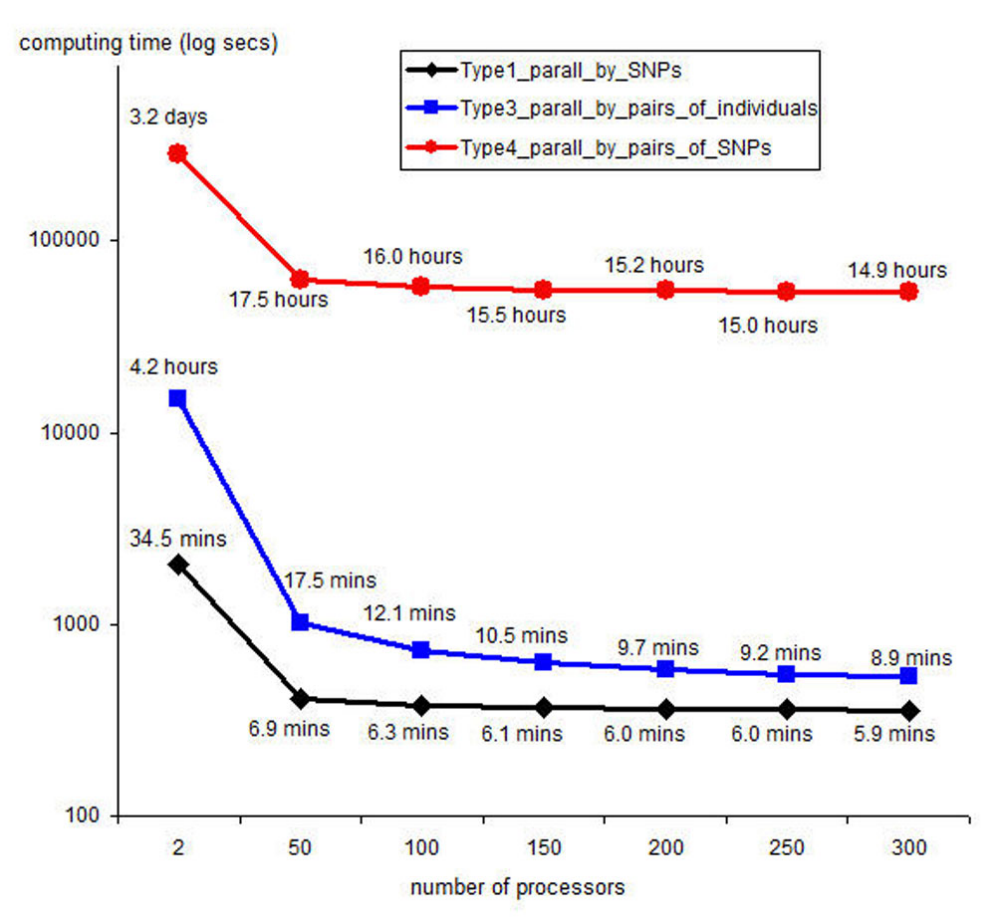
**The computing time on a large cluster**. The computing time on a large cluster for Type1_parall_by_SNPs, Type3_parall_by_pairs_of_individuals and Type4_parall_by_pairs_of_SNPs extrapolated from Figure 7 applying the overhead equation.

If the number of available processors is *P*, the parallel computing time for *P *processors is *time_P_cpus*, and the serial computing time for a processor is *time_a_cpu*; the overhead for *P *processors is:

Different numbers of processors produce different overheads depending on data partitioning, network communicating, and data merging. However, the overheads can be predicted based on the overhead of eight processors shown in Figure 7. The computing time on a large cluster for Type1_parall_by_SNPs, Type3_parall_by_pairs_of_individuals and Type4_parall_by_pairs_of_SNPs extrapolated from Figure 7 applying the above overhead equation are shown in Figure 8. It is clear that ParallABEL also saves the computing time on a large cluster. In addition, the time-saving rates for these types are much increased when the number of processors is between 2 and 50. Nevertheless, the time-saving rates are slowly increased when the number of processors is greater than 100. This applies to the particularly and relatively small data set analyzed here. With bigger data sets, the time-saving rates can be larger. However, the user should optimize the number of processors according to the gain in computational throughput.

## Discussion and conclusions

We have presented the ParallABEL library which employs parallel computing to reduce computing time for data intensive tasks. ParallABEL can run on clustered computers that support LAM/MPI and R. With clustered computers, processors or even personal computers can be easily added as new compute-nodes. ParallABEL runs on both distributed and shared memory architectures as it was developed with MPI. For a distributed memory architecture, MPI usually uses a computer network for task communications. For a shared memory architecture, MPI does not employ the network for task communications. This means that a distributed memory architecture may exhibit more overhead than a shared memory architecture (for example, eight single-core processors versus a single eight-core processor). In our experiments, Type1_parall_by_SNPs took only 6 minutes to execute on a shared memory architecture but 14 minutes on a distributed memory architecture. The overhead of a shared memory architecture was tested on a server, which has 2 QUAD-CORE Intel Xeon(R) (2.8 GHz) processors and 8 GB. The server runs on CentOS version 5.4, and provides Open MPI version 1.4.1.

ParallABEL allows the user to specify the number of processors employed for data execution. We expect computational performance to increase linearly with the number of processors when using Type1_parall_by_SNPs, Type3_parall_by_pairs_of_individuals, and Type4_parall_by_pairs_of_SNPs. In addition, ParallABEL is faster than GenABEL on one processor. Computing times for Type3_parall_by_pairs_of_individuals and Type4_parall_by_pairs_of_SNPs are longer than those for Type1_parall_by_SNPs because the input data consists of pairs of individuals and SNPs respectively, which are much larger than the SNPs input for Type1_parall_by_SNPs. In addition, if the number of SNPs is n, then the number of inputs for Type1_parall_by_SNPs is n but the number of inputs data for Type4_parall_by_pairs_of_SNPs is n*n. ParallABEL can save much more computational time when utilizing Type3_parall_by_pairs_of_individuals and Type4_parall_by_pairs_of_SNPs than when using Type1_parall_by_SNPs. Therefore, as the amount of input data increases, the time saved by ParallABEL also increases. ParallABEL does not only reduce the computing time but also is as easy-to-use as the more conventional GenABEL.

ParallABEL can not reduce the computing time when the data size is too small, such as the result shown when employing the hom function of Type2_parall_by_individuals, because the computing time is too short. In that case, the overheads of data partitioning and output merging overwhelm the computational performance.

## Availability and requirements

• **Project home page:**

http://www.sci.psu.ac.th/units/genome/CGBR/ParallABEL/index.html

http://parallabel.r-forge.r-project.org/

• **Operating system(s): **Platform independent

• **Programming language: **R

• **Other requirements: **LAM/MPI or Open MPI, Rmpi, GenABEL

• **License: **GPL for non-profit organizations

• **Any restrictions to use by non-academics: **license needed

## Authors' contributions

All authors conceived and designed the project. PT provided the Hanuman Cluster. US, SM and YSA implemented the software. US conducted computational performance evaluation. All authors drafted, read, revised, and approved the manuscript.
